# Supplementary material for: Size Exclusion Chromatography–Mass Photometry: A New Method for Adeno-Associated Virus Product Characterization
Source: Cells. 2023 Sep 13;12(18):2264. doi: 10.3390/cells12182264 (PMC10528216; doi:10.3390/cells12182264)
Supplement: Supplementary file 1 [file cells-12-02264-s001.zip › cells-2562885-supplementary.pdf]

SEC-MP: A New Method for Adeno-Associated Virus Product Characterization

Supplementary Materials:

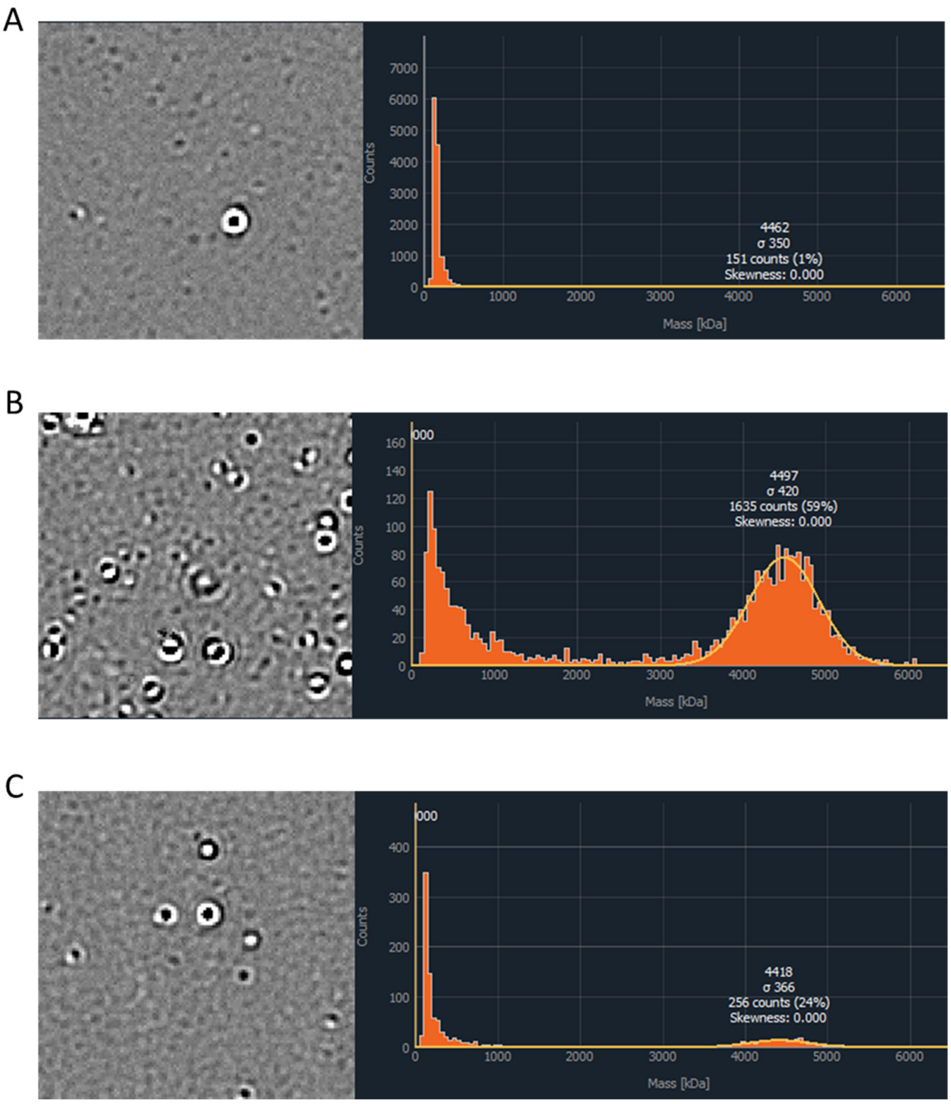

Figure S1: Full-width MP mass distributions of AAV samples. Left panels show representative frames from MP measurements. Right panels show the mass distribution histograms obtained from the respective MP measurements. (A) MP analysis of a AAV sample at a low loading concentration. The mass distribution shows a significant presence of molecules in the 0 to 500 kDa range, constituting approximately 99% of the total histogram counts.(B) MP analysis of the same sample in (A) at a higher loading concentration. Detection of small molecules becomes challenging due to image background noise, manifested as smaller peaks appearing in the 0 – 500 kDa range. The detection of AAV particles in this sample is not significantly affected, since the AAV peak Gaussian fit parameter are comparable with the result shown in panel (A). (C) MP analysis of the same AAV sample after SEC purification. The loading concentration is similar to data shown in panel (A). Despite the SEC purification, a noticeable amount of small impurities is still present. In comparison to (A), the fraction of AAV particles increases to 24%. In terms of the AAV detection, the data quality in (A) and (C) is comparable.

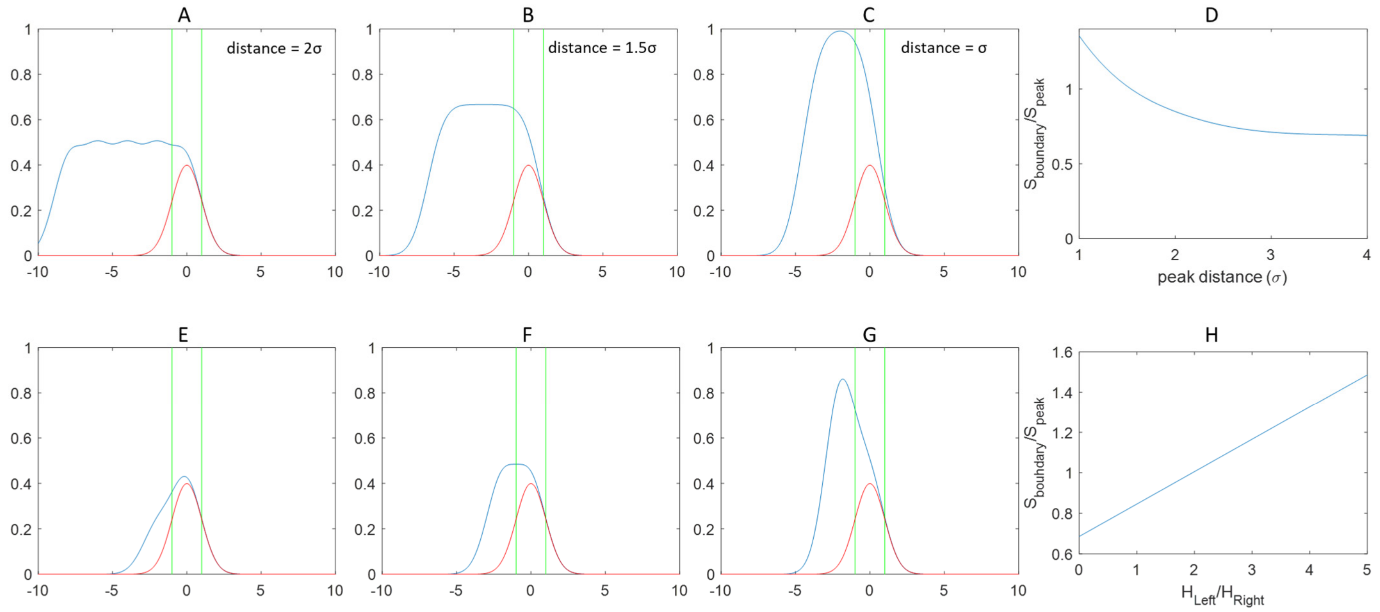

Figure S2: Simulation of the errors introduced by peak boundary setting. Top row – the effect of the peak separation: In panel (A) to (C), blue curves represent the sum of 5 identical Gaussian peaks, where the distances between adjacent peaks are 2σ, 1.5σ and 1σ, from left to right panel, respectively. The red curves represent the right-most Gaussian peak of these sums. The green lines represent the peak boundaries separated by 2σ. Panel (D) shows the ratio of the area under the blue curve integrated within the boundaries to the whole area under the red peak. Bottom row – the effect of peak size: In panel (E) to (G), blue curves represent the sum of 2 Gaussian peaks separated by 2σ. The ratios of left peak height to the right peak height (H_{Left}/H_{Right}) are 0.5, 1, and 2 from left to right, respectively. The red curves represent the Gaussian peak positioned on the right side of the pair. The green lines represent the integration boundaries separated by 2σ. Panel (H) shows the ratios of the area under the blue curve within the boundaries to the whole area under the red peak. This ratios shown in panel (D) and (H) provide an estimation of the integration errors resulting from the placement of the boundaries.
